# Supplementary material for: Antibody-mediated feedback modulates interclonal competition in the germinal center
Source: bioRxiv. 2025 Nov 7:2025.11.06.686519. Preprint. [Version 1] doi: 10.1101/2025.11.06.686519 (PMC12637549; doi:10.1101/2025.11.06.686519)
Supplement: 1 [file NIHPP2025.11.06.686519V1-supplement-1.pdf]

bioRxiv preprint doi: https://doi.org/10.1101/2025.11.06.686519; this version posted November 7, 2025. The copyright holder for this preprint (which was not certified by peer review) is the author/funder, who has granted bioRxiv a license to display the preprint in perpetuity. It is made available under aCC-BY 4.0 International license.

SUPPLEMENTAL FIGURES AND LEGENDS

1 ACCTCCATGGTGGAGAAGGAGATTCTAGCTGTGGTCAGAAAAGAGAAAGAAGAAACCAGTCTGAAAGTGTCTTTGCAAAGAAACATGGGGAACGGCCTCC

101 TCTCCTCAGGGTGCAGCCTCTATGAGTCATCGGACCTGTCCCTCATGAAGTTGCCTCACAGCAACCCACTACCTCTGGTGCCTGTAAAGGTCAAACAAGA

201 GACTGTTGAGCCCATGGATCCGGGTGGAGGATCGGGTATAACTTCGTATAGCATACATTATACGAAGTTATCGTAGTGATAAGATACATTGATGAGTTTG

301 GACAAACCACAACTAGAATGCAGTGAAAAAAATGCTTTATTTGTGAAATTTGTGATGCTATTGCTTTATTTGTAACCATTATAAGCTGCAATAAACAAGT

401 TAACAACAACAATTGCATTCATTTTATGTTTCAGGTTCAGGGGGAGATGTGGGAGGTTTTTTAATAACTTCGTATAGCATACATTATACGAAGTTATCTG

501 GAAGCGGCGCCACTAATTTCAGTCTTCTGAAACAGGCCGGAGACGTGGAGGAGAATCCTGGACCCACCATGAAGCTGCTGCCGTCGGTGGTGCTGAAGCT

601 CCTTCTGGCTGCAGTTCTTTCGGCACTGGTGACTGGCGAGAGCCTGGAGCAGCTTCGGAGAGGGCTAGCTGCTGGAACCAGCAACCCGGACCCTTCCACT

701 GGATCTACGGACCAGCTGCTACGCCTAGGAGGCGGCCGGGACCGGAAAGTCCGTGACTTGCAAGAGGCAGATCTGGACCTTTTGAGAGTCACTTTATCCT

801 CCAAGCCACAAGCACTGGCCACACCAAGCAAGGAGGAGCACGGGAAAAGAAAGAAGAAAGGCAAGGGACTAGGGAAGAAGAGGGACCCATGTCTTCGGAA

901 ATACAAGGACTTCTGCATCCACGGAGAATGCAAATATGTGAAGGAGCTCCGGGCTCCCTCCTGCATCTGCCACCCAGGTTACCATGGAGAGAGGTGTCAT

1001 GGGCTGAGCCTCCCAGTGGAAAATCGCTTATATACCTATGACCATACAACTATCCTGGCTGTGGTGGCCGTGGTGCTGTCCTCTGTCTGTCTGCTGGTCA

1101 TCGTGGGGCTTCTCATGTTTAGGTACCATAGGAGAGGTGGTTATGATGTGGAAAACGAAGAGAAAGTGAAGTTGGGCATGACTAATTCCCACTAAGATTT

1201 TCAGAAAATAAGTGTTTCGTGTTGCTTCTTAGGGTATGGCTTGGTGAATCAGGGTGCCTTTAGCAAATTGCTTGTACATGACTCCAGATCTGCAAAGCTC

1301 CGCTGGCACCGGGTGCTTCCCTGCACCTCTCTGGAATTAAAGAAGGACTCCAATGTTACCAAAATCTCAGGGCATAAATGAGGCAAAGACTC

Underline, homology arms
Cyan, *Prdm1* terminal exon
Green, GGGS linker
Yellow, loxP sites
Grey, SV40 polyadenylation sequence
Magenta, P2A peptide
Red, DTR (*Chlorocebus sabaeus* HB-EGF)
Orange, *Prdm1* 3'UTR

Figure S1. Annotated sequence for the repair oligo used to build the *Prdm1*$^{LSL\text{-}DTR}$ allele.

bioRxiv preprint doi: https://doi.org/10.1101/2025.11.06.686519; this version posted November 7, 2025. The copyright holder for this preprint (which was not certified by peer review) is the author/funder, who has granted bioRxiv a license to display the preprint in perpetuity. It is made available under aCC-BY 4.0 International license.

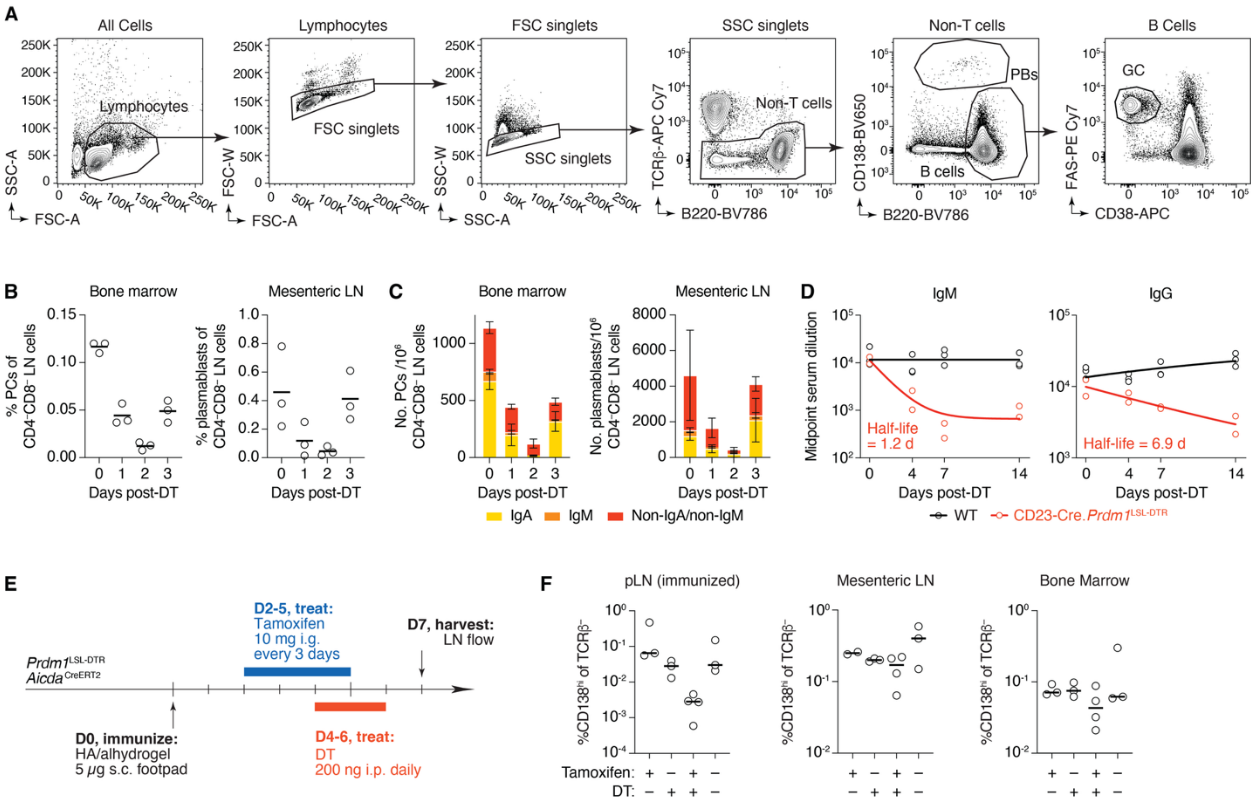

Figure S2. Characterization of plasma cell and antibody depletion in *Prdm1*$^{\text{LSL-DTR}}$ mice. **(A)** Gating strategy for identification of LN plasmablasts and GC B cells. In some experiments, GC B cells were identified as CD38^{low} GL-7^{+}, and in some experiments PCs were identified as CD4/CD8^{-}CD138^{hi}. **(B)** Kinetics of depletion and resurgence of bone marrow PCs and mesenteric LN plasmablasts following i.p. administration of 200 ng of DT to CD23-Cre.*Prdm1*$^{\text{LSL-DTR}}$ mice. Each symbol represents one mouse. **(C)** As in (B), but following the counts per million LN cells of PCs and plasmablasts of each isotype. Non-IgA/non-IgM cells are presumably IgG^{+}, given that the latter isotype is downregulated upon B cell differentiation to the plasmablast and PC fate. Error bars are S.D. for three mice as in (B). **(D)** Cre.*Prdm1*$^{\text{LSL-DTR}}$ mice were given 200 ng of DT daily for the duration of the experiment and serum samples were collected at 0, 4, 7, and 14 days post-treatment. Midpoint serum titers of total IgM and IgG were determined by ELISA and the half-life of each isotype was determined using one-phase exponential decay. Each symbol represents one mouse, the line shows the fitted exponential curve. (**E**) Experimental setup. (**F**) Quantification of LN plasmablasts (*left and middle*) and bone marrow PCs (*right*) 7 days post HA immunization depending on treatment regimens as indicated below graphs. Each symbol represents one mouse. Results are pooled from two independent experiments with n=1-2 mice per group. Bars represent medians.

bioRxiv preprint doi: https://doi.org/10.1101/2025.11.06.686519; this version posted November 7, 2025. The copyright holder for this preprint (which was not certified by peer review) is the author/funder, who has granted bioRxiv a license to display the preprint in perpetuity. It is made available under aCC-BY 4.0 International license.

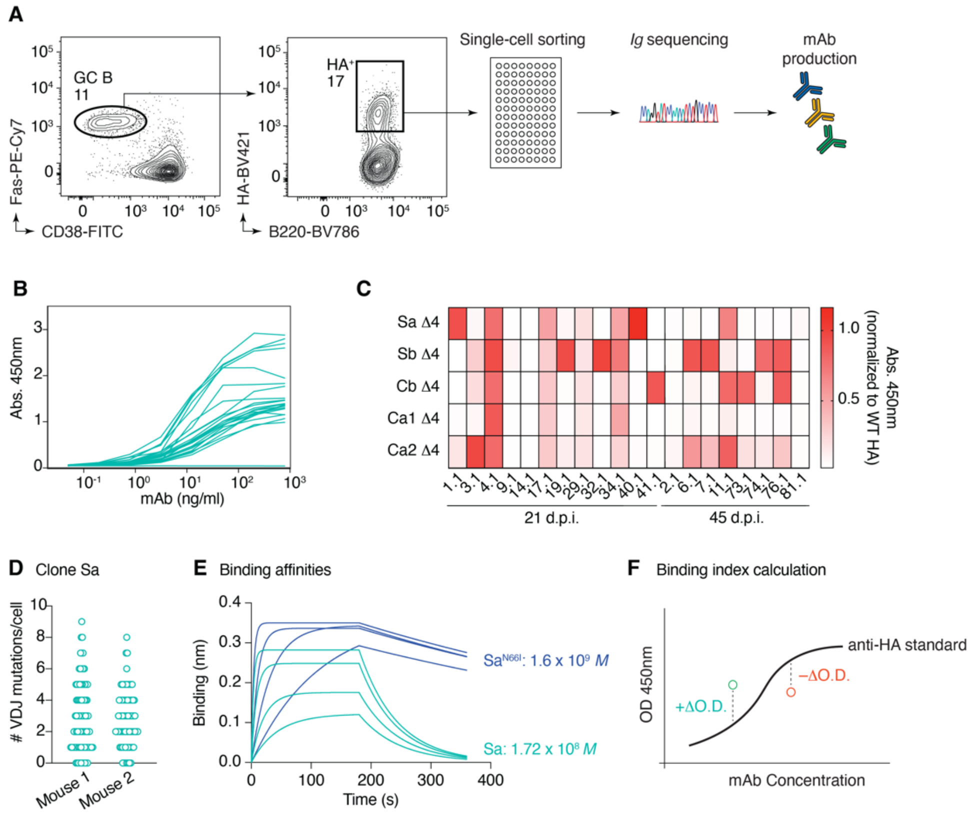

Figure S3. Characterization of monoclonal antibodies specific for influenza HA. (A) C57BL/6 mice were infected with PR8 influenza virus and mediastinal GC B cells were assayed by flow cytometry 21 and 45 days later. HA tetramer binding B cells were single cell sorted, their *Ighv* and *Igkv* loci were sequenced, and monoclonal antibodies were produced from members of 20 expanded clones. **(B)** Titration curves of candidate mAbs binding to recombinant PR8 HA by ELISA. **(C)** ELISA of mAbs binding to five Δ4 mutants, in which all but one of the five antigenic sites of PR8 HA are mutated. mAbs were assayed at a concentration of 1 μg/ml. Sequences for (A) and (C) are available in Supplemental Spreadsheet 1. **(D)** Number of *Igh* nucleotide mutations per cell in Ig^{Sa} GC B cells 18 dpi with HA. **(E)** Binding kinetics curves of Sa sequence and N66I mutation Fabs as measured by bio-layer interferometry. Fabs were assayed at concentrations of 160, 120, 80 and 40 nM. **(F)** Schematic demonstrating calculation of binding index. Black curve represents binding of reference mAb Cb to HA in ELISA. mAbs with better binding than Cb will be higher than the curve (green circle), while mAbs with decreased binding will be lower (red circle).

bioRxiv preprint doi: https://doi.org/10.1101/2025.11.06.686519; this version posted November 7, 2025. The copyright holder for this preprint (which was not certified by peer review) is the author/funder, who has granted bioRxiv a license to display the preprint in perpetuity. It is made available under aCC-BY 4.0 International license.

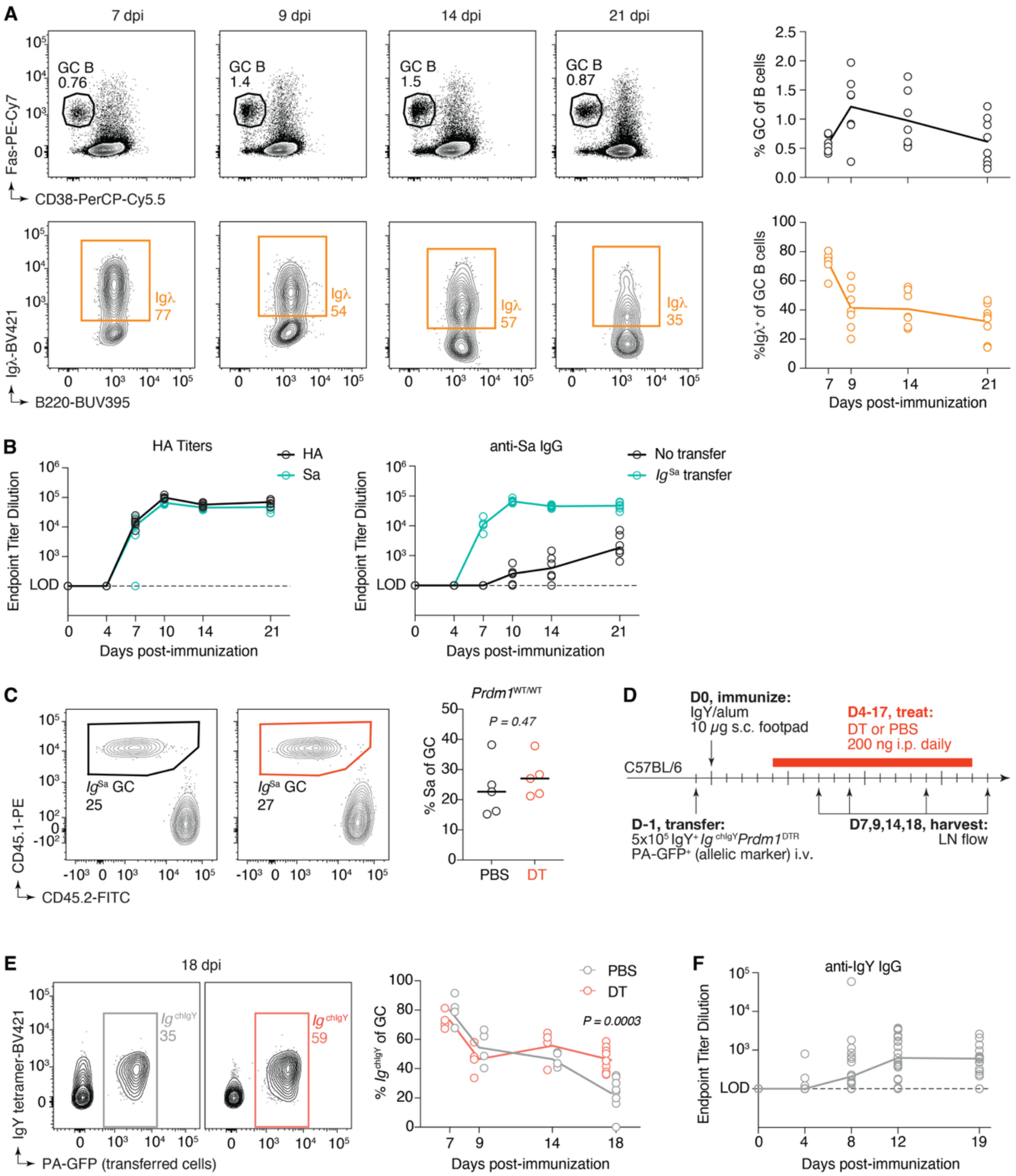

Figure S4. Antibody mediated feedback is not limited to the Sa epitope. (A) Immunization time course with NP-OVA following Igλ^{+} GC B cells in C57BL/6 mice. Top row shows representative flow cytometry plots and quantification of GC size and bottom row shows representative flow cytometry plots and quantification of Igλ^{+} GC occupancy. **(B)** Sa-specific and total HA IgG1 titers in C57BL/6 mice following *Ig*^{Sa} transfer and HA immunization. **(C)** Same experimental setup as in 4D, but *Ig*^{Sa} cells do not carry the *Prdm1*^{DTR} allele. Shown are representative flow cytometry plots (*left*) and a quantification (*right*) of *Ig*^{Sa} GC B cells 18 dpi. **(D)** Experimental setup for panel (E). **(E)** Representative flow cytometry plots and fraction of *Ig*^{chIgY} GC B cells at 7,9,14 and 18 dpi with IgY. PAGFP-transgenic mice are used as a marker for flow cytometry, without photoactivation. **(F)** IgY-specific IgG1 titers in C57BL/6 mice following *Ig*^{chIgY} transfer and IgY immunization. (A,D) Results are pooled from 2 independent experiments, with n = 2-4 mice per group. (B) Data are pooled from two independent experiments, with n = 3 mice per group. (C) Data are pooled from two independent experiments, with n = 2-3 mice per group. (F) Data are pooled from two independent experiments, with n = 8-11 mice per group. (B,F) Lines connect medians. In all plots, each symbol represents one mouse. P-values are for Student's T-test.

bioRxiv preprint doi: https://doi.org/10.1101/2025.11.06.686519; this version posted November 7, 2025. The copyright holder for this preprint (which was not certified by peer review) is the author/funder, who has granted bioRxiv a license to display the preprint in perpetuity. It is made available under aCC-BY 4.0 International license.

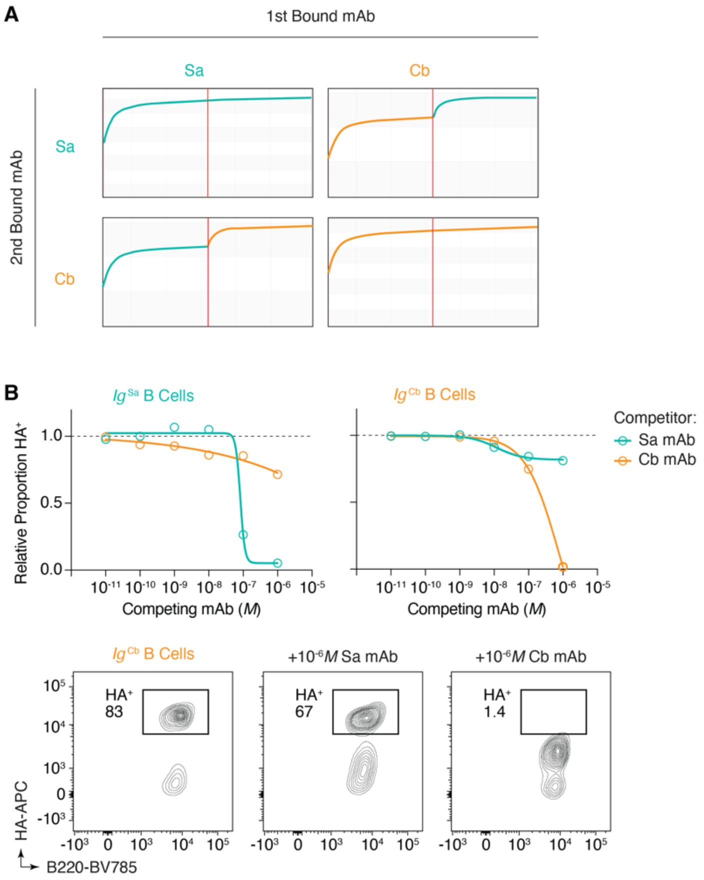

Figure S5. Characterization of two HA-specific monoclonal antibodies with non-overlapping epitopes. (A) Epitope binding via bio-layer interferometry. Binding of one mAb to PR8 HA was followed by binding with another, and binding to distinct sites is indicated by continued mass accumulation onto the sensor. **(B)** Flow-cytometry competition assays between Ig^{Sa} or Ig^{Cb} B cells and soluble mAbs for binding to HA tetramer. mAbs were co-incubated with tetramer at increasing concentrations, and inhibition of B cell staining was assessed. Proportion of HA^{+} is normalized to binding of HA in the absence of competing antibody.
